# Metronidazole-induced encephalopathy

**DOI:** 10.4102/sajr.v25i1.2016

**Published:** 2021-03-18

**Authors:** Vikash G. Lala, Bilal Bobat, Mark Haagensen, Prakash Kathan, Adam Mahomed

**Affiliations:** 1 Department of Gastroenterology, Faculty Internal Medicine, Charlotte Maxeke Johannesburg Academic Hospital, Johannesburg, South Africa; 2 Department of Gastroenterology, Faculty Internal Medicine, Wits Donald Gordan Medical Centre, Johannesburg, South Africa; 3 Department of Radiology, Faculty of Health Sciences, Wits Donald Gordan Medical Centre, Johannesburg, South Africa; 4 Department of Neurology, Faculty of Health Sciences, Wits Donald Gordan Medical Centre, Johannesburg, South Africa

**Keywords:** metronidazole, metronidazole-induced cerebellar ataxia, metronidazole-induced neurotoxicity, metronidazole adverse events, metronidazole-induced encephalopathy, dentate nucleus lesions, splenium lesions, corpus callosum lesions

## Abstract

Metronidazole is a widely used antibacterial and antiprotozoal agent for a number of conditions. Whilst its more common gastrointestinal side effects are well known, neurotoxicity remains under-recognised. Both central and peripheral neurological side effects have been described. This report describes a case of radiologically confirmed metronidazole-induced cerebellar ataxia in a cirrhotic patient with a review of the literature. Awareness of this side effect is essential for prompt recognition as early drug withdrawal leads to resolution in the majority of cases.

## Introduction

### Background

Metronidazole is commonly used as an antibacterial and antiprotozoal agent belonging to the nitroimidazole class of antibiotics, which is particularly useful in the treatment of anaerobic infections.^1^ Furthermore, it is frequently used in the management of hepatic encephalopathy,^2,3,4^ *Clostridioides* (formerly clostridium) *difficile*-associated diarrhoea,^5,6^ *Helicobacter pylori* eradication,^7^ suppurative complications of inflammatory bowel disease (IBD) and in the prevention of the postoperative re-occurrence of IBD.^8,9^ The adverse effects of metronidazole include nausea, vomiting, diarrhoea, abdominal cramping, anorexia, hypersensitivity, a metallic taste and a disulfiram-type reaction with ethanol. Neurological adverse effects include a peripheral neuropathy, optic neuropathy, autonomic neuropathy, seizures, encephalopathy and cerebellar toxicity.

## Case presentation

A 68-year-old male, with no prior comorbidities, was referred to our institution with hepatic cirrhosis for further management and evaluation for liver transplantation. His work-up revealed the most likely aetiology for his cirrhosis to be a combination of alcoholic and non-alcoholic fatty liver disease. Whilst under our care he was admitted with decompensated cirrhosis manifested by hepatic encephalopathy, and responded favourably to treatment with lactulose and rifaximin. After being discharged, he subsequently presented with a week history of progressive ataxia and imbalance. He admitted to self-medicating with metronidazole; however, the exact cumulative dose and duration of treatment remained unknown.

He described that the ataxia fluctuated during the day but, in general, was worsening. Upon clinical examination, he appeared to be generally well with voluntary ptosis and slurred speech. He had an intention tremor and mild dysdiadochokinesia with severe ataxia. He was considered to have an acute onset of ataxia, which was thought to be most likely because of drugs or toxins. Wernicke’s encephalopathy was excluded. Inflammatory and structural causes were considered as a differential diagnosis, which prompted us to image the brain with magnetic resonance imaging (MRI).

**FIGURE 1:**
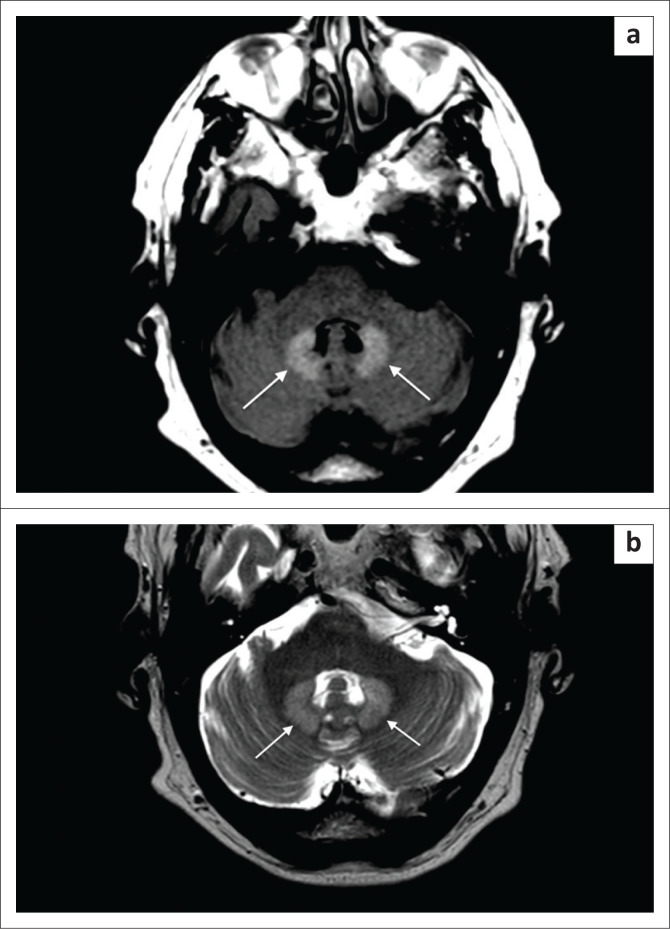
Fluid attenuated inversion recovery (a) and T2 turbospin echo sequence (b) demonstrating high signal intensity in the dentate nuclei bilaterally (white arrows).

**FIGURE 2:**
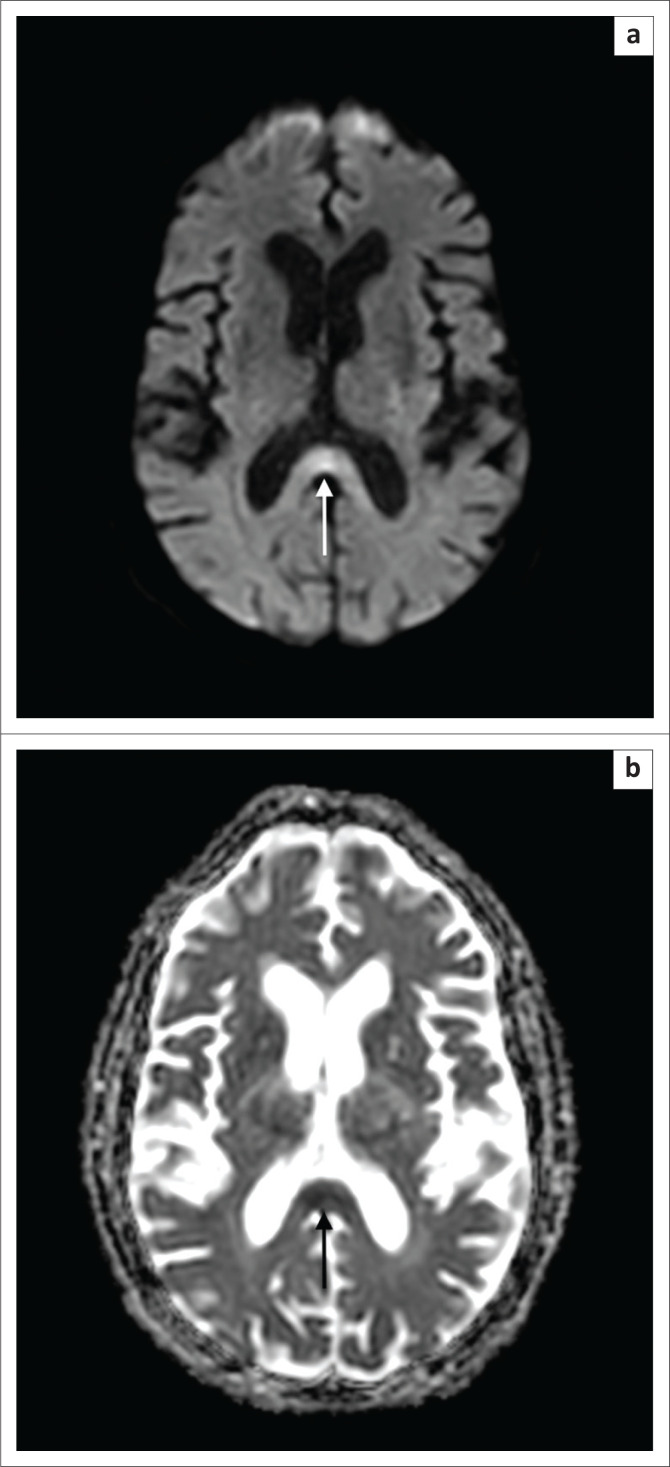
Diffusion-weigthed imaging (a) indicating increased signal in the splenium of the corpus callosum and corresponding apparent diffusion coefficient map (b) showing a decreased signal in this area in keeping with restriction.

The MRI brain sequences included T2 turbospin echo, fluid attenuated inversion recovery (FLAIR) and echo-planar diffusion-weighted sequences, which all showed increased signal in the dentate nuclei (Figure 1). These changes were not seen on a previous MR brain scan, obtained 3 weeks earlier for non-specific headaches. The diffusion signal in the dentate nuclei showed no restriction on the apparent diffusion coefficient (ADC) map and was suggestive of vasogenic oedema. Diffusion signal was, however, also noted in the splenium of the corpus callosum, which showed no discernible T2 or FLAIR signal, and in this location there was evidence of diffusion restriction with low signal on the ADC map suggesting cytotoxic oedema (Figure 2). No abnormal enhancement was noted postcontrast. No signal change was observed in the posterior pons or midbrain, which has been described previously in cases of metronidazole toxicity.^10^

**FIGURE 3:**
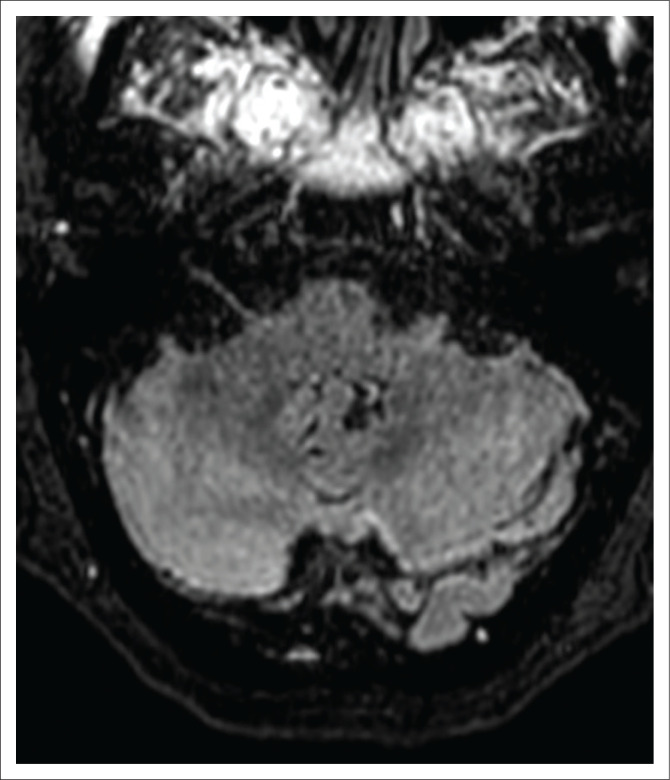
Fluid attenuated inversion recovery imaging at the 7-week follow-up imaging, demonstrating resolution of the previous dentate nuclei high signal; compare with Figure 1.

**FIGURE 4:**
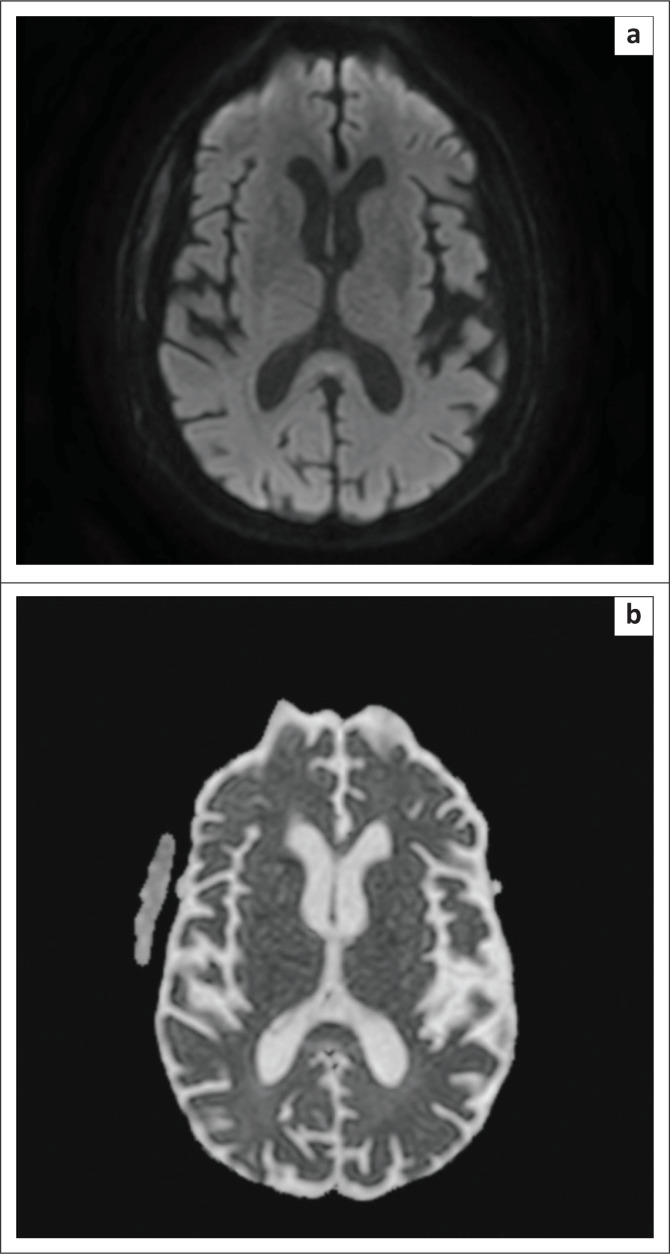
(a) Diffusion sequence showing resolution of the splenium lesion at follow up (b) Apparent diffusion coefficient map showing complete resolution of the splenium lesion at follow up imaging; compare with Figure 2.

A follow-up scan approximately 7 weeks later (Figures 3–4), after cessation of metronidazole, showed complete clearing of the previous signal in the dentate nuclei and splenium of the corpus callosum. No interval atrophy of the splenium, cerebral hemispheres or cerebellum was observed. This was mirrored by clinical resolution of his signs and symptoms.

## Discussion

The role of antibiotics in the management of hepatic encephalopathy has been described with the proposed mechanism of effect being a reduction in gut-derived neurotoxins, endotoxins and inflammation. Antimicrobial agents used in the management of hepatic encephalopathy include metronidazole, neomycin, vancomycin, paromomycin and rifaximin.^11^ Metronidazole has been shown to be equally efficacious to neomycin in small studies^2^ and a newer study has shown comparable efficacy to rifaximin in acute hepatic encephalopathy.^4^ Its use, however, is limited by its side effect profile, which may be compounded by its reduced elimination amongst patients with cirrhosis.^12^ Despite this, it is still commonly used, particularly in resource-limited settings where access to newer agents may be limited.

Metronidazole-induced neurotoxicity is likely an under-recognized and under-reported entity, with its true incidence remaining unknown. Despite this, a number of case reports documenting both metronidazole-induced peripheral neuropathies^13^ and encephalopathy^14,15,16,17,18^ have been published. A recent systematic review identified 136 patients with metronidazole-induced encephalopathy.^19^ In this study, encephalopathy was reported across a wide range of age groups; although, most case studies were documented in patients above the age of 40. The average cumulative dose amongst these patients was found to be 125.7 g. However, a broad range in the cumulative dose was noted, with the lowest documented dose being as low as 5 g. Similarly, the duration of metronidazole used also varied from 2 days to several years.^19^ In contrast, smaller literature reviews suggested that neurotoxicity was related to the prolonged use of metronidazole and high cumulative doses.^20^ The total cumulative dose in our patient remains unknown but prolonged use of metronidazole was reported. Amongst patients with metronidazole-induced peripheral neuropathies, most had received a cumulative dose of > 42 g in a systematic review.^13^

Whilst the exact pathogenetic mechanism underlying the development of encephalopathy remains unknown, several mechanisms have been hypothesised. Early work postulated the binding of metronidazole or metabolites to ribonucleic acid (RNA) in neurons with the resultant degeneration of axons.^21^ Furthermore, the generation of superoxide radicals leading to myelin oedema and vacuolation is thought to be contributory.^22^ Other proposed mechanisms include focal ischaemia and the possible role of mitochondrial dysfunction.^23^

Most patients with metronidazole-induced encephalopathy present with cerebellar signs, the most frequent being dysarthria, gait instability and ataxia.^19^ Patients may less commonly present with changes in mental state, vertigo, dizziness, seizures and lateralising signs.^19,20^ Other conditions such as Wernicke’s encephalopathy, toxic encephalopathies, other drug encephalopathies, enteroviral encephalomyelitis, hepatic encephalopathy and some leukodystrophies should be considered in the differential diagnosis.

Radiological imaging plays an important role in the diagnosis of metronidazole-induced encephalopathy. Furthermore, imaging studies may be useful in excluding other causes of neurological dysfunction. Magnetic resonance imaging changes are typically bilateral and symmetrical, with hyperintensities on T2-weighted images in the cerebellar dentate nuclei being the most commonly involved area.^19,10^ Other locations commonly involved include the splenium of the corpus callosum, the midbrain, the pons and the medulla. Involvement of splenium with T2-weighted hyperintensities warrants the exclusion of other conditions, such as epilepsy, Marchiafava–Bignami disease, acute disseminated encephalitis, infective encephalitis, extra-pontine myelinolysis, systemic lupus erythematosus, renal failure and vitamin B12 deficiency.^23^ The subcortical white matter and basal ganglia are less commonly involved.^19,10^ The findings on diffusion-weighted imaging (DWI) and the ADC vary according to the anatomical areas involved, suggesting cytotoxic oedema in some areas and vasogenic oedema in others.^19,10^

The majority of patients with metronidazole-induced encephalopathy have clinical resolution within 2 weeks of drug cessation.^16,18,19,20^ In addition, the majority of patients with clinical resolution have some degree of radiological improvement. Radiological resolution upon drug withdrawal may thus further support the diagnosis. A small percentage of patients (4%) had documented residual neurological deficits in a systematic review.^19^ Amongst these patients, the total cumulative dose was not related to residual symptomatology; however, all were found to have white matter hyperintensities on the initial MRI. Failure of radiological improvement may be related to the anatomical areas involved and in instances with a low ADC and diffusion restriction.^23^ Death was uncommon (5%) and unlikely to be primarily attributed to metronidazole-induced encephalopathy, with most of the deceased having significant premorbid conditions.^19^

## Conclusion

The true incidence of metronidazole-induced neurotoxicity remains unknown. Despite under-recognition, the disease is rare and largely confined to case reports, case series and systematic reviews. To our knowledge, this is the first documented case report in South Africa. Metronidazole is a commonly used antimicrobial agent especially in resource constraint areas where access to other newer agents is limited. Awareness of this important side effect is essential for its early diagnosis and management. The importance of recognising the condition early is emphasised by the resolution of disease, in the majority of cases, with drug cessation. Metronidazole remains to be an effective therapeutic agent, which should be judiciously prescribed where indicated and cautiously used in those with significant co-morbid illnesses.
